# Selenium and the risk of cancers of the colon, pancreas and stomach

**DOI:** 10.1186/1897-4287-10-S3-A13

**Published:** 2012-04-20

**Authors:** Marcin Lener, Anna Wiechowska-Kozłowska, Józef Kładny, Magdalena Muszyńska, Grzegorz Sukiennicki, Lidia Kubera-Nowakowska, Jan Lubiński

**Affiliations:** 1 Department of Genetics and Pathology, Pomeranian Medical University in Szczecin, Poland; 2 Laboratory of Endoscopy, Division of Health Care Ministry of Internal Affairs and Administration in Szczecin, Poland; 3 Department of General and Oncological Surgery, Pomeranian Medical University, Szczecin, Poland

## Introduction

Research suggests that selenium may influence the behavior of the cancer risk in two ways. As an antioxidant, selenium helps to protect the body against free radicals. Selenium may also prevent or slow tumor growth, as some breakdown products of selenium can inhibit tumor growth by enhancing immune cell activity and inhibition of tumor blood vessel development.

## Aim

The aim of this study was to determine the level of selenium in blood serum as a potential marker of risk for cancers of the colon, stomach or pancreas.

## Material and methods

The research material was a total of 94 samples of blood serum from people with cancer, diagnosed and confirmed in one of the organs: colon (55 cases), pancreas (30 cases) or stomach (9 cases) and 94 samples of blood serum derived from healthy individuals which paired control group. The criteria adopted for pairing included: gender, year of birth (+/- 3 years), history of the occurrence of cancers in the family among first degree relatives and smoking status expressed in pack-years.

Selenium concentration in blood plasma was determined using graphite furnace atomic absorption spectrometry (GFAAS). The measurement accuracy was +/- 5% µg Se/l.

## Results

Association between Se concentration and frequency of cancers in quartiles are presented in table 1. Statistical analyses are summarized in table 2.

**Table 1:** Association between Se plasma concentration and risk of cancers analyzed.

Cancer site	Quartile	Se concentration range (µg/l)	Cases [% - no]
Pancreas	I	25,69-50,09	100% (15/15)

	II	50,72-65,58	60% (9/15)

	III	66,34-73,30	40% (6/15)

	IV	74,07-113,89	- (0/15)

Colorectal and stomach	I	15,92-55,25	87,5% (28/32)

	II	56,08-67,05	59,37% (19/32)

	III	67,22-75,96	25% (8/32)

	IV	76,45-103,36	28,13% (9/32)

**Tab.2:** Results of statistical analyses of cancer site depending on Se concentration.

Cancer site	Quartiles compared	Se concentration range (µg/l)	Cases/controls in compared groups	Fisher’s exact test		
	
				P	OR	CI
Pancreas	I vs II	25,69-50,09 vs 50,72-65,58	15/0 vs 9/6	0,017	21,2	1,07-421,11

	I vs III	25,69-50,09 vs 66,34-73,30	15/0 vs 6/9	0,0007	45,3	2,2-899,53

	I vs IV	25,69-50,09 vs 74,07-113,89	15/0 vs 0/15	<0,0001	961	7,9-51,617

Colorectal and stomach	I vs II	15,92-55,25 vs 56,08-67,05	(5)*28/4 vs (3)*19/13	0,0219	4,79	1,35-16,94

	I vs III	15,92-55,25 vs 67,22-75,96	(5)*28/4 vs (0)*8/24	<0,0001	21	5,6-78,5

	I vs IV	15,92-55,25 vs 76,45-103,36	(5)*28/4 vs (1)*9/23	<0,0001	17,9	4,8-65,7

*stomach cancer cases

## Conclusions

The obtained results suggest that low levels of selenium in the body may correlate with an increased risk of pancreatic cancer, colon or stomach, and thus constitute one of the markers of risk for cancers of such sites. Research requires the extension to a larger number of samples including tumor size, and performance analysis for selenoprotein genes.

Prospective studies can elucidate:

a) the use of selenium measurements as markers of risk of above cancers;

b) possibility of lowering risk of the cancers of the colon, pancreas and stomach by supplementation of diet with selenium.

